# The microprotein encoded by exosomal lncAKR1C2 promotes gastric cancer lymph node metastasis by regulating fatty acid metabolism

**DOI:** 10.1038/s41419-023-06220-1

**Published:** 2023-10-30

**Authors:** Ke-Gan Zhu, Jiayu Yang, Yuehong Zhu, Qihang Zhu, Wen Pan, Siyu Deng, Yi He, Duo Zuo, Peiyun Wang, Yueting Han, Hai-Yang Zhang

**Affiliations:** 1 Tianjin Medical University Cancer Institute and Hospital, National Clinical Research Center for Cancer, Key Laboratory of Cancer Prevention and Therapy, Tianjin’s Clinical Research Center for Cancer, Tianjin Medical University, Tianjin, 300060 China; 2 Department of Pathology, College of Basic Medicine, Chongqing Medical University, Chongqing, 400016 China

**Keywords:** Cancer metabolism, Transport carrier

## Abstract

Lymph node metastasis (LNM) is the prominent route of gastric cancer dissemination, and usually leads to tumor progression and a dismal prognosis of gastric cancer. Although exosomal lncRNAs have been reported to be involved in tumor development, whether secreted lncRNAs can encode peptides in recipient cells remains unknown. Here, we identified an exosomal lncRNA (lncAKR1C2) that was clinically correlated with lymph node metastasis in gastric cancer in a VEGFC-independent manner. Exo-lncAKR1C2 secreted from gastric cancer cells was demonstrated to enhance tube formation and migration of lymphatic endothelial cells, and facilitate lymphangiogenesis and lymphatic metastasis in vivo. By comparing the metabolic characteristics of LN metastases and primary focuses, we found that LN metastases of gastric cancer displayed higher lipid metabolic activity. Moreover, exo-lncAKR1C2 encodes a microprotein (pep-AKR1C2) in lymphatic endothelial cells and promotes CPT1A expression by regulating YAP phosphorylation, leading to enhanced fatty acid oxidation (FAO) and ATP production. These findings highlight a novel mechanism of LNM and suggest that the microprotein encoded by exosomal lncAKR1C2 serves as a therapeutic target for advanced gastric cancer.

## Introduction

Gastric cancer ranks the fifth among all the malignancies in the world, with nearly one million new cases each year [1]. More than half of the gastric cancer patients are found to have lymph node metastasis (LNM) at the initial diagnosis, which is closely related to poor prognosis and recurrence [2, 3]. LNM is the most important factor for the overall survival of gastric cancer patients following curative gastrectomy, and the increased number of metastatic lymph nodes often indicates a decrease in survival rates [4–7]. Metastasis to lymph nodes also leads to the subsequent metastasis to other organs and the development of cancer [8–10], and illuminating the mechanisms underlying LNM is an urgent problem to solve.

Tumor cells always balances the processes of material and energy synthesis to adapt to unfavourable conditions [11]. Studies have shown that abnormal lipid metabolism plays an important role in the occurrence and development of tumors, and the lipid metabolism of gastric cancer and other tumor cells has undergoes substantial changes [12–14]. Fatty acid oxidation (FAO) comprises of a cyclical series of reactions, which result in the shortening of fatty acids and generate important metabolites, including NADPH, FADH2 and acetyl-CoA [11]. The oxidation of fatty acids can provide twice as much energy as carbohydrates, and tumor cells can improve the level of fatty acid oxidation by increasing lipid uptake and fatty acid synthesis In order to improve the efficiency of energy supply [15, 16]. Carnitine palmitoyltransferase 1 A (CPT1A) has been reported to be the key enzyme of FAO, and is upregulated in a cancers [17–19]. Although targeting key genes involved in lipid metabolism and fatty acid oxidation has been demonstrated to inhibit the progression of solid tumors [20, 21], the role of lipid metabolism in lymphangiogenesis and lymph node metastasis in gastric cancer has not been reported.

Exosomes belong to the superfamily of extracellular vesicles (EVs), with diameters ranging from 30–150 nm [22, 23]. Exosomes are secreted from all cell types and are well known to mediate the signal communication between cells by delivering lncRNAs, circRNAs, proteins, lipids and DNAs. Mounting evidence has shown that exosomes are involved in each stage of tumor development, including tumor growth, immune escape, angiogenesis, distant metastasis and drug resistance [24–26]. Recent studies have shown that certain lncRNAs and circRNAs containing open reading frames (ORFs) encode short peptides [27–29], but whether exosome delivered lncRNAs or circRNAs can produce peptides in target cells has not yet been reported. Therefore, the molecular basis of lymphangiogenesis and lymph node metastasis regulated by GC secreted exosomal lncRNAs deserves further study and exploration.

Herein, we report that an exo-lncRNA (ENST00000604184, termed lncAKR1C2 because it shares the same maternal gene as AKR1C2) was upregulated in the serum of gastric cancer patients with LNM, and was positively related to lymphatic vessel density in a VEGFC-independent manner. Subsequently, we identified a 163 amino acid microprotein (pep-AKR1C2) in lymph endothelial cells, which was encoded by GC derived exosomal lncAKR1C2. The lncAKR1C2-encoded microprotein was demonstrated to enhance FAO and ATP production in lymph endothelial cells by regulating YAP-mediated CPT1A expression, resulting in the promotion of lymphangiogenesis and LNM. Metabonomic analysis also confirmed higher lipid metabolism levels in lymph node metastases than in the primary focus. Our findings illustrate a novel mechanism of LNM mediated by exo-lncRNA encoded microprotein and provide potential targets for the clinical treatment of gastric cancer.

## Results

### Exosomal lncAKR1C2 positively correlated with GC lymph node metastasis

**Fig. 1:**
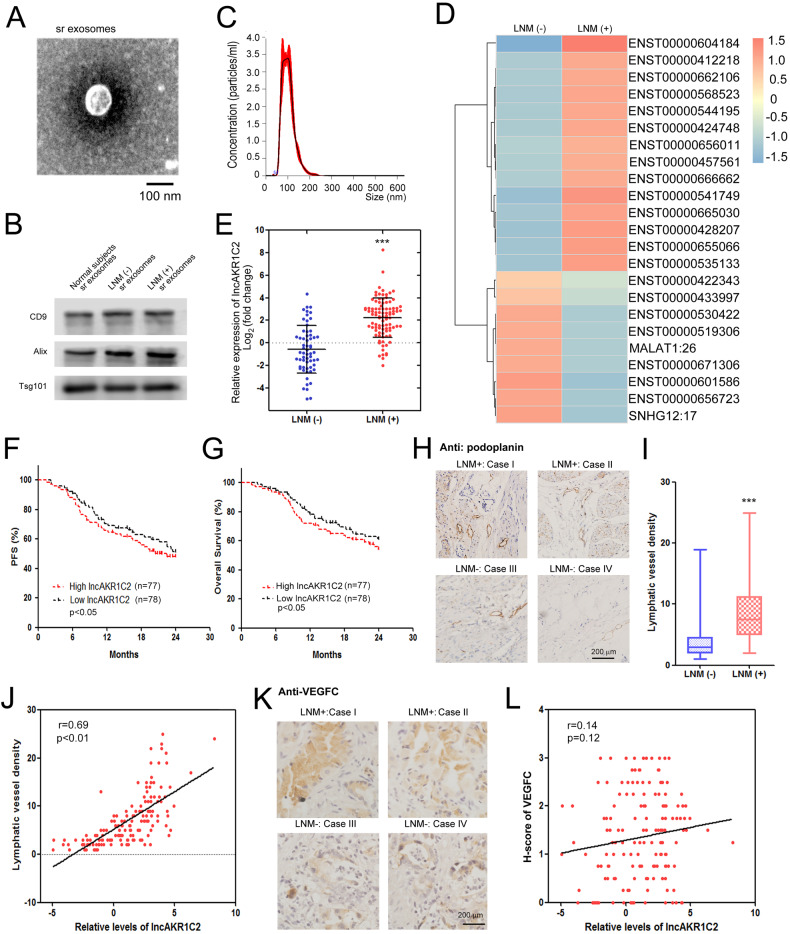
Exosomal lncAKR1C2 is related to GC lymphatic metastasis. **A**, **B** Serum exosomes isolated from GC patients were identified by TEM (**A**) and Nano-Tracking Analysis (**B**). **C** Western blot analysis of exosomal protein markers in purified serum exosomes. **D** The serum exosomal lncRNA expression patterns of GC patients with (LNM + , *n* = 48) or without lymphatic metastasis (LNM-, *n* = 28). **E** RT-qPCR analysis of lncAKR1C2 in the tumor tissues of the LNM(-) (*n* = 61) and LNM(+) (*n* = 94) groups. High expression of lncAKR1C2 is associated with poor PFS (**F**) and OS (**G**) in GC (*n* = 155). H. IHC analysis of podoplanin in both the LNM(-) (*n* = 61) and LNM(+) (*n* = 94) groups. **I** Quantitative analysis of (**H**). **J** lncAKR1C2 is positively linked with lymphatic vessel density (*n* = 155). K. IHC analysis of VEGFc in both LNM(-) (*n* = 61) and LNM(+) (*n* = 94) groups. **L** lncAKR1C2 has no obvious correlation with VEGFC (*n* = 155). ***indicates p < 0.001.

To determine the exosomal lncRNAs related to LNM, serum exosomes were isolated from stage II/III GC patients before surgical operation and lncRNA-seq was performed. Exosomes of both the LNM- and LNM+ groups were observed by using transmission electron microscopy (Fig. 1A), and exosome markers, including CD9, Tsg101 and Alix were detected by WB analysis (Fig. 1B). The size of the exosomes was determined by nanotracking analysis, and the diameters of these serum exosomes were approximately 30–150 nm (Fig. 1C). High-throughput sequencing results revealed that a list of exo-lncRNAs were significantly changed in the LNM+ group (*n* = 48) compared with patients without LNM (*n* = 28), and the lncRNA ENST00000604184 (lncAKR1C2) showed the highest increase (Fig. 1D). The results of further tests on an expanded sample confirmed that exo-lncAKR1C2 was upregulated by more than 8-fold in the LNM+ group (*n* = 94) compared with the LNM- group (*n* = 61) (Fig. 1E). A total of 155 GC patients were divided into two groups based on the median number of lncAKR1C2 expression. Kaplan-Meier analysis showed that high levels of lncAKR1C2 predicted shorter progression free survival (PFS) (Fig. 1F) and overall survival (OS) (Fig. 1G).

Lymphangiogenesis is directly related to LNM in various types of cancer, and exosomes and non-coding RNA have been proven to be involved in the process [30–33]. The IHC analysis using anti-podoplanin antibody indicated that the density of lymph vessels was much higher in patients with LNM (Fig. 1H, I), and lncAKR1C2 levels showed a positive link with lymphatic vessel density (Fig. 1G). Although VEGFC was also upregulated in GC patients with LNM (Fig. 1K), there was no significant correlation between VEGFC and lncAKR1C2 (Fig. 1L). These results suggested that exosomal lncAKR1C2 is correlated with LNM in gastric cancer, and this process might be independent of VEGFC.

### Ribo-Seq analysis indicates that lncAKR1C2 has the potential to encode a microprotein

**Fig. 2:**
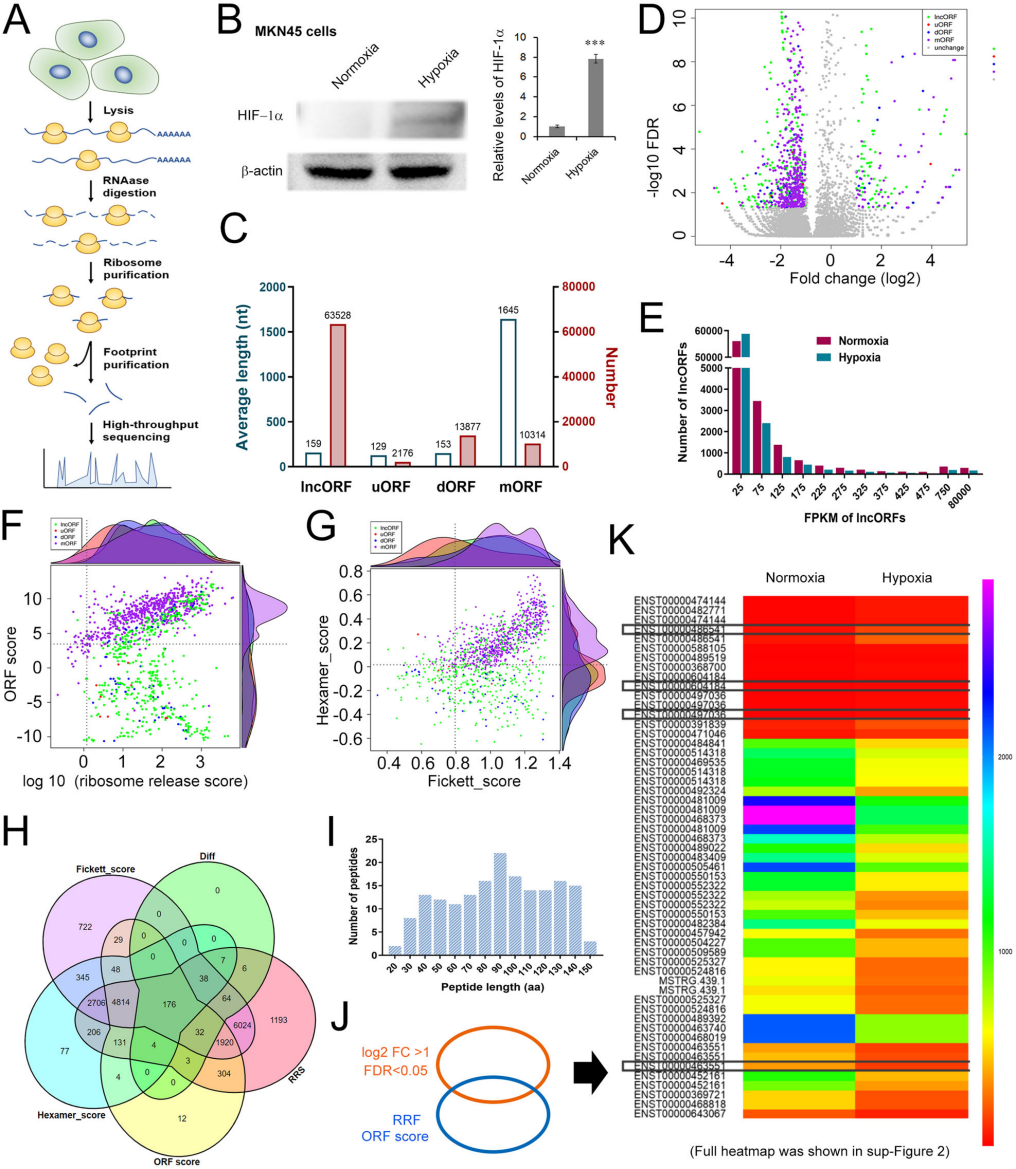
Screening of hypoxia-induced lncRNAs with coding function by translational omics. **A** Schematic representation of the principles of Ribo-seq. **B** Western blot analysis of HIF-1α in normoxia and hypoxia treated MKN45 cells (*n* = 3). **C** Average lengths and numbers of identified lncORFs, uORFs, dORFs, and mORFs by Ribo-seq. **D** The volcano plot of all kinds of discrepant sORFs between groups by Ribo-seq. E. The expression and distribution of lncORFs in both groups. The ORF score (**F**) and Hexamer_score (**G**) of all kinds of sORFs. **H** Venn diagram of the lncRNAs with high–translation potential under hypoxic conditions. **I** Leng distribution of encoded short peptides. **J** Comprehensive analysis of differential expression and bioinformatics for identified lncORFs. **K** Heatmap of specific lncRNAs in both groups. ***indicates p < 0.001.

We next analysed the characteristics of lncAKR1C2 using translation omics. The flow diagram of Rio-seq is shown in Fig. 2A. As hypoxia is one of the common features of solid tumors, and we compared the differences in lncRNA translation omics between gastric cancer cells under hypoxic and normoxic conditions. Hypoxia inducible factor-1α (HIF-1α) was significantly upregulated in MKN45 cells under hypoxic treatment (Fig. 2B). In addition to the main protein-coding ORF (mORF), some sORFs less than 300 nucleotides (nt) from RNAs that were traditionally considered unable to encode proteins were also identified, including lncORFs from lncRNAs, uORFs from upstream regions of mRNAs and dORFs from downstream regions of mRNAs. As expected, the lengths of lncORFs, uORFs and dORFs were much shorter than those of mORFs, while the number of lncORFs was more than sixfold that of mORFs (Fig. 2C). The fold change and false discovery rate (FDR) of these ORFs were shown in Fig. 2D, and a group of lncORFs were obviously increased. In addition, lncRNA ORFs with a fold change (FC) ≥ 2 and false discovery rate (FDR) < 0.05 were screened using the edgeR package (http://www.r-project.org/). Read counts in ORFs were calculated, and the translation levels were normalized by the fragments per kilobase of transcript per million mapped reads (FPKM) method. The levels of lncORFs in both groups were detected, and the majority of all identified lncORFs were less than 125 FPKM (Fig. 2E), and the length of peptides ranged from 30–140 aa (Fig. 2I). The ORF score and RRS score (ribosome release score) are shown in Fig. 2F, and the Fickett score and Hexamer score are shown in Fig. 2G. A total of 176 lncORFs were screened out by synthetically using multiple scoring systems (Fig. 2H). The main part of the heatmap displaying the expression of these 176 lncRNAs were shown in Fig. 2J and Fig. 2K, and the full heatmap is shown in Figure S1. It was found that lncAKR1C2 (ENST00000604184) was closely bound to the ribosome, suggesting that lncAKR1C2 has the potential to encode a microprotein.

### Detection of lncAKR1C2-encoded microprotein in gastric cancer cells

**Fig. 3:**
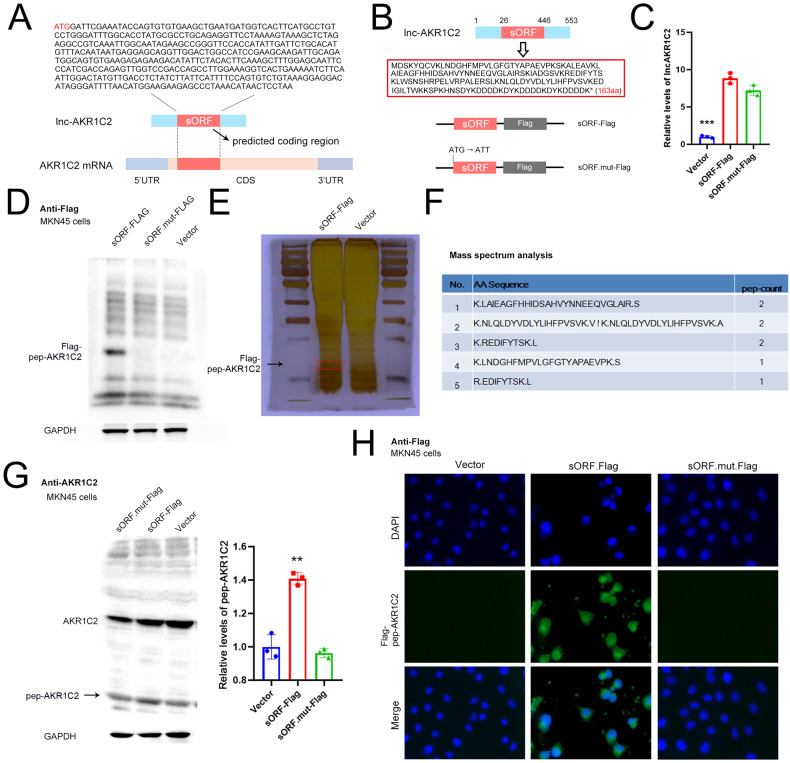
Detection of the microprotein encoded by lncAKR1C2. **A** The predicted binding region (sORF) in lncAKR1C2. **B** Schematic diagram of Flag fusion constructs of wild-type (sORF-Flag) and mutant lncAKR1C2 (sORF.mut-Flag). The start codon of the sORF was mutated to ATT. **C** Relative levels of lncAKR1C2 in MKN45 cells transfected with sORF-Flag or sORF.mut-Flag (*n* = 3). **D** WB analysis of Flag fusion microprotein (Flag-pep-AKR1C2) in MKN45 cells by using anti-Flag antibody. **E**, **F** Silver staining (**E**) combined with mass spectrum analysis (**F**) for pep-AKR1C2. G. WB analysis of Flag fusion microprotein (Flag-pep-AKR1C2) by using anti-AKR1C2 antibody (*n* = 3). **H** The expression and intracellular localization of Flag-pep-AKR1C2 in MKN45 cells detected by immunofluorescence. ** indicates p < 0.01 and *** indicates p < 0.001.

Four lncRNAs with the potential to encode short peptides based on the Ribo-Seq analysis were selected for the subsequent validation. To detect the expression of lncRNA-encoded peptides, the plasmids containing full sequences of the four lncRNAs, as well as a Flag-tag at the c-terminus of ORFs were constructed. The WB analysis using an anti-Flag antibody confirmed that ENST00000486541 and ENST00000604184 (lncAKR1C2) have the ability to encode short peptides (Fig. S2). The predicted coding region (sORF) in lncAKR1C2 as well as in the AKR1C2 mRNA is shown in Fig. 3A, and the CDS region of AKR1C2 mRNA covers the full length of the sORF. The Ribo-Seq data indicated that lncAKR1C2 has the potential to encode a 163-aa short microprotein (termed pep-AKR1C2), thus a lncAKR1C2-overexpressing plasmid with the mutated initial codon (ATG → ATT) was also constructed (Fig. 3B). Both the wild-type (sORF-Flag) and mutant lncAKR1C2 (sORF.mut-Flag) caused an increase in lncAKR1C2 RNA levels (Fig. 3C), but the Flag-labelled pep-AKR1C2 was detected only in the sORF-Flag group (Fig. 3D). We used silver staining combined with mass spectrometry (MS) to analyse pep-AKR1C2, the amino acid sequences of the fragments detected by MS were consistent with the prediction of Ribo-Seq (Fig. 3E, F). As pep-AKR1C2 is part of the host gene (AKR1C2), pep-AKR1C2 expression could also be detected by using an anti-AKR1C2 antibody (Fig. 3G). Finally, the distribution of pep-AKR1C2 was determined by immunofluorescence (IF), and pep-AKR1C2 was mainly found in the cytoplasm (Fig. 3H). Taken together, these results indicated that lncAKR1C2 encodes a 163-aa microprotein in gastric cancer cells.

### Exosome delivered lncAKR1C2 encodes a microprotein in lymphatic endothelial cells

**Fig. 4:**
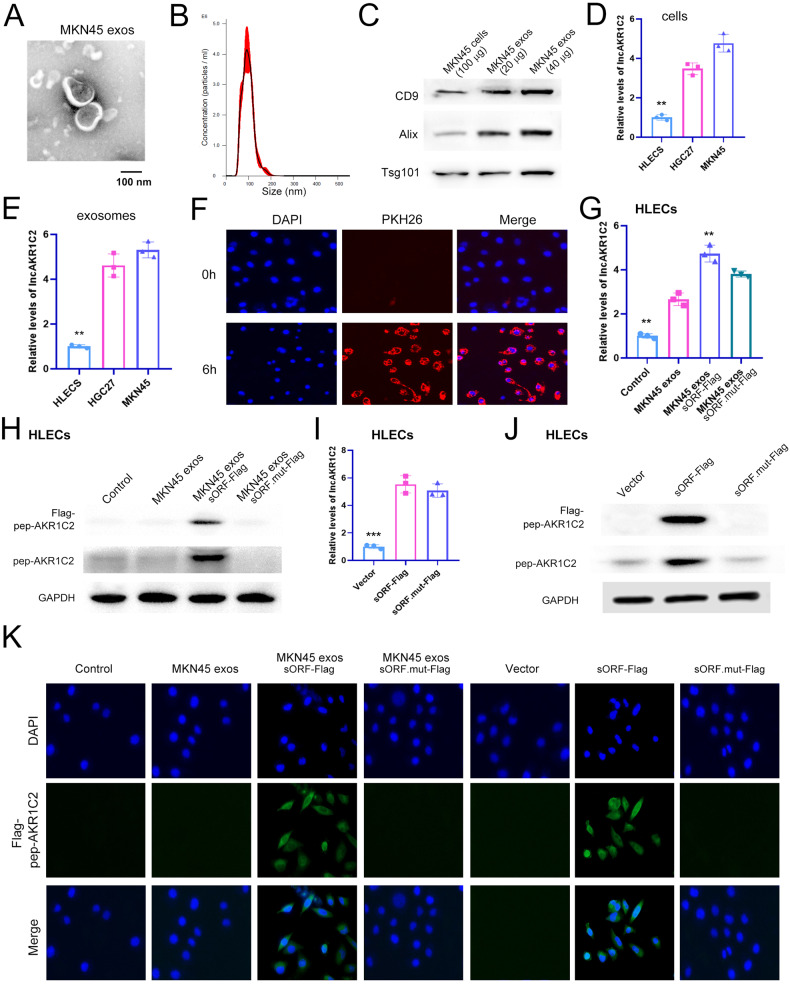
GC secreted exo-lncAKR1C2 can encode a microprotein in lymphatic endothelial cells. **A**, **B** Exosomes of MKN45 cells were identified by TEM (**A**) and Nano-Tracking Analysis (**B**). C WB analysis of CD9, Alix and Tsg101 in purified MKN45 exosomes and MKN45 cells. **D** Relative levels of lnc-AKR1C2 in GC cell lines (MKN45 and HGC27) and human lymphatic endothelial cells (HLECS) (*n* = 3). **E** Relative levels of lnc-AKR1C2 in the exosomes of MKN45, HGC27 and HLECS cells (*n* = 3). **F** The fusion of PKH26-labelled MKN45 exos with HLECS. **G**, **H** HLECS were treated with MKN45 exos, MKN45 exos sORF-Flag and MKN45 exos sORF.mut-Flag, and lnc-AKR1C2 levels (**G**) and Flag-pep-AKR1C2 (**H**) were detected. **I**, **J** HLECS were transfected with sORF-Flag, sORF.mut-Flag or control vector, and lnc-AKR1C2 levels (I) and Flag-pep-AKR1C2 (J) were detected. **K** Representative images of immunofluorescence (IF) by HLECS treated as described above. ** indicates p < 0.01 and *** indicates p < 0.001.

Data from clinical analysis have demonstrated that exosomal lncAKR1C2 is linked with lymphangiogenesis and LNM, and we next tested the expression of pep-AKR1C2 encoded by GC secreted lncAKR1C2 in human lymphatic endothelial cells (HLECS). The exosomes derived from MKN45 cells were observed by transmission electron microscopy, NTA and WB analysis using exosome markers, and the diameters of most exosomes were approximately 100 nm (Fig. 4A–C). By comparing lncAKR1C2 levels in GC cells and HLECS, we found that lncAKR1C2 was markedly overexpressed in both GC cells and GC exosomes (Fig. 4D, E). MKN45 cells were overexpressed with wild-type lncAKR1C2 or mutant lncAKR1C2 respectively, and exosomes were collected. These exosomes were labelled with PKH26 and used to coculture with HLECS, and the fusion of MKN45 exosomes and HLECs was observed within 6 h (Fig. 4F), resulting in the significant upregulation of lncAKR1C2 in HLECS (Fig. 4G). As is shown in Fig. 4H, Flag-pep-AKR1C2 was detected only in the HLECS cells treated with MKN45 exosomes containing wild-type sORF-Flag, and pep-AKR1C2 is increased in the MKN45 exos sORF-Flag group, suggesting that exosomal lncAKR1C2 can encode a microprotein in HLECS cells. To provide direct evidence for pep-AKR1C2 in lymphatic endothelial cells, HLECS were transfected with wild-type lncAKR1C2, mutant lncAKR1C2 or blank vector. The results of WB analysis showed the same effect as those of the exosome treated groups (Fig. 4I, J). Moreover, IF analysis also indicated that pep-AKR1C2 mainly exists in the cytoplasm of HLECS cells (Fig. 4K). Therefore, these data demonstrated that GC-secreted exo-lnAKR1C2 can encode a microprotein in lymphatic endothelial cells and pep-AKR1C2 likely functions in cytoplasm.

### Lymphangiogenesis requires an exo-lncAKR1C2 dependent metabolic shift towards FAO

**Fig. 5:**
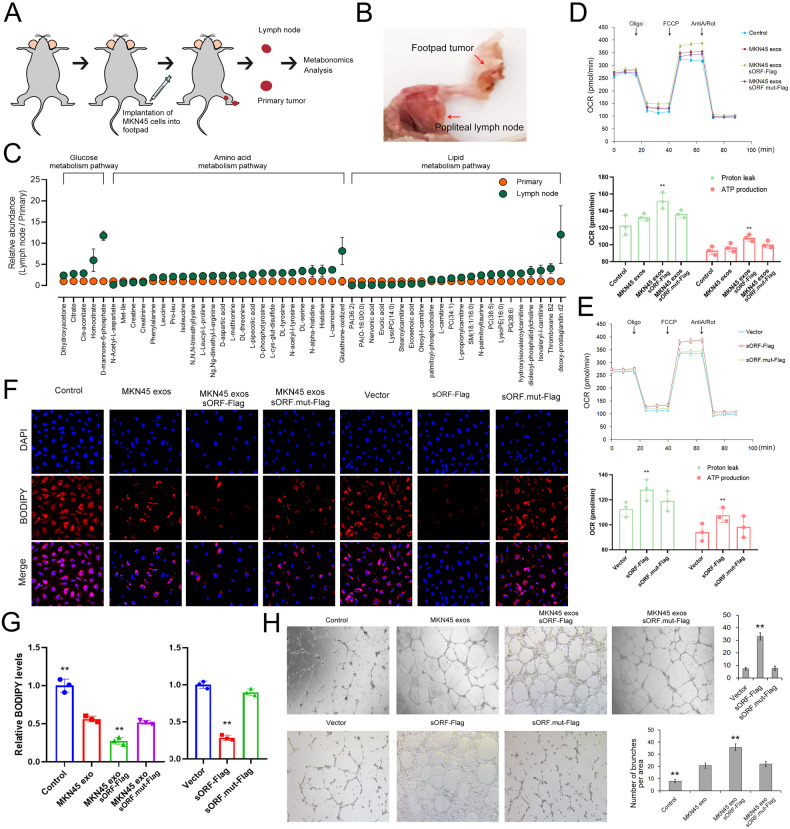
Exosomal lncAKR1C2 promotes lymphangiogenesis in vitro by enhancing fatty acid utilization. **A** Flow diagram of metabolic analysis for the primary focuses and lymph node metastases isolated from mice with tumor-implantation in footpad. **B** Representative histogram analysis of primary focuses and lymph node metastases. **C** Comparison of metabolome profiles between primary focuses (implanted in footpad) and lymph node metastases (*n* = 6). **D**, **E**. Oxygen consumption rates (OCRs) were quantified under basal conditions and with drugs that disrupt the respiratory chain using a Seahorse Biosciences XF 96 analyser in HLECS cells treated with saline, MKN45 exos, MKN45 exos sORF-Flag and MKN45 exos sORF.mut-Flag, or transfected with sORF-Flag, sORF.mut-Flag and control vector (*n* = 3). **F**. BODIPY staining (dyes of fatty acids) of HLECS cells (*n* = 3). G. Quantitative analysis of (**F**). **H** Tube formation of HLECS cells treated as described above (*n* = 3). ** indicates p < 0.01.

Previous studies have shown that LN metastatic tumors exhibit increased fatty acid accumulation and oxidation by using a melanoma footpad implantation model [14]. Here, we also determined the metabolic characteristics of GC lymph node metastasis by combining a GC footpad implantation model and metabolomics analysis (Fig. 5A, B). The comparison of metabolome profiles between primary GC tumor (implanted in the footpad) and LN metabolic tumors is shown in Fig. 5C, and the higher levels of lipid metabolism were observed in LN metastasis. Furthermore, the metabolic rate of HLECS was determined by using Seahorse Biosciences XF96 analyser. As is expected, lymphatic endothelial cells treated with GC exosomes or lncAKR1C2 showed a higher oxygen consumption ratio (OCR) from proton leakage and an increase in the maximal respiratory capacity (Fig. 5D, E). However, the mutated lncAKR1C2 had little influence on the OCR of HLECS.

To assess the role of exosomal lncAKR1C2 in fatty acid metabolism, HLECS cells were treated with oleic acid (OA) for 24 h and then cultured them in low glucose media for an additional 48 h to allow sufficient utilization of fatty acids. It was revealed that MKN45 exosomes and lncAKR1C2 significantly promoted the utilization of OA and ATP production in HLECS cells, especially in the MKN45 exosomes overexpressing wild-type lncAKR1C2 (Figs. 5F, G and S3). To conclude, LN metastatic GC tumors showed higher lipid metabolism activity than primary foci, which requires secreted exosomal lncAKR1C2.

The number of rings formed by HLECS cells and the number of migrated cells increased in the MKN45 exos group, and were largest with the treatment of MKN45 exosomes overexpressing with wild-type lncAKR1C2. Transfection of lncAKR1C2 also showed similar effects (Figs. 5H and S4). These results demonstrated that GC secreted lncAKR1C2 promotes tube formation and migration of lymphatic endothelial cells.

### LncAKR1C2 encoded microprotein promotes CPT1A expression via the YAP pathway in lymphatic endothelial cells

**Fig. 6:**
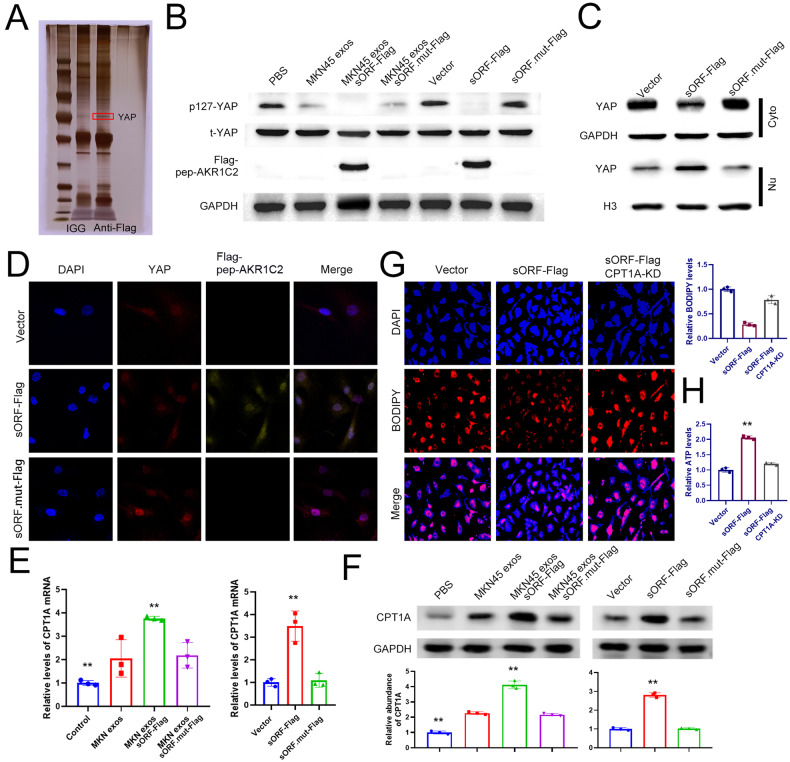
Pep-AKR1C2 promotes the expression of FAO-related CPT1A by decreasing YAP phosphorylation. **A** Silver staining analysis of the proteins interacting with pep-AKR1C2. **B** Pep-AKR1C2 encoded by exo-lncAKR1C2 decreases p127-YAP levels in HLECs cells. **C** Pep-AKR1C2 promotes the nuclear translocation of YAP. **D** Immunofluorescence colocalization analysis was used to determine the interaction between YAP and pep-AKR1C2. **E**, **F** Determination of CPT1A mRNA (**E**) and protein levels (**F**) in HLECS treated as above. G. Knockdown of CPT1A rescues the high-consumption level of fatty acids mediated by overexpressed lnc-AKR1C2 (*n* = 3). **H** Knockdown of CPT1A rescues the high ATP production levels mediated by overexpressed lnc-AKR1C2 (*n* = 3). ** indicates p < 0.01.

To explore the potential mechanism of pep-AKR1C2 mediated fatty acid metabolism and lymphangiogenesis, co-IP combined with LC-MS/MS was performed to identify the proteins interacting with pep-AKR1C2. The 63 proteins detected by mass spectrometry are shown in Extended Table 1, including YAP (Yes-associated protein), one of the core proteins in the Hippo pathway (Fig. 6A). Recent studies have reported that YAP is involved in lymph node metastasis of tumors by regulating metabolic reprogramming in cancer cells [14, 34, 35]; however, the role of YAP in lymph endothelial cells remains unclear. The direct interaction between YAP and pep-AKR1C2 (labelled with Flag) was validated by co-IP and WB analysis (Fig. S5). It was shown that MKN45 exosomes and lncAKR1C2 had little effect on total YAP expression (t-YAP), but notably decreased phosphorylated YAP levels (p127-YAP) (Fig. 6B), which in turn promoted the entry of YAP into the nucleus (Fig. 6C). Subsequently, the direct interaction between YAP and pep-AKR1C2 was also validated by immunofluorescence co-localization analysis (Fig. 6D). Considering that the relationship between YAP and CPT1A has been reported in recent studies [34], we also checked the expression of CPT1A in HLECS cells. GC secreted exosomal lncAKR1C2 significantly upregulated CPT1A mRNA and protein levels (Fig. 6E, F). Moreover, the effects of lncAKR1C2 on fatty acid utilization and ATP production were proven to be dependent on CPT1A (Fig. 6G, H). Together, these data suggest that pep-AKR1C2 encoded by exo-lncAKR1C2 promotes FAO-related CPT1A expression by decreasing YAP phosphorylation.

### Exosomal lncAKR1C2 promoted lymphatic metastasis in vivo

**Fig. 7:**
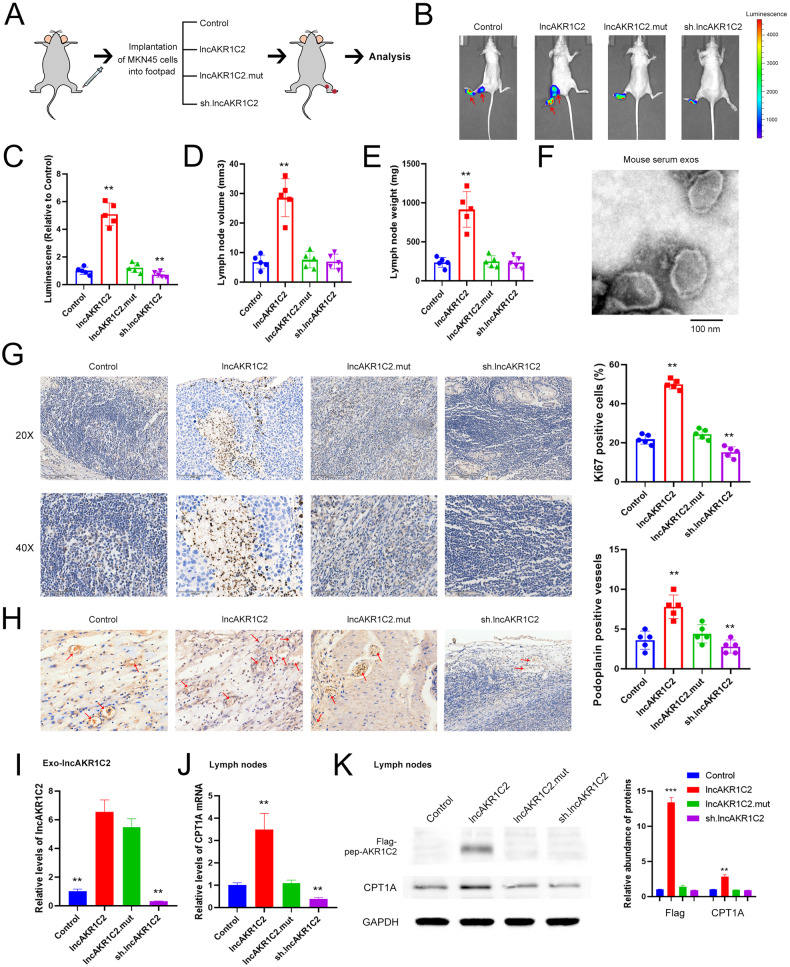
In vivo role of exo-lncAKR1C2 in promoting gastric cancer lymphatic metastasis. **A** Schematic description of tumor implantation into footpad. **B**, **C** Histogram analysis of tumors in footpad and lymph nodes (*n* = 5). **D**, **E** The volumes (D) and weights (E) of lymph nodes (*n* = 5). **F** Representative image of mouse serum exosomes identified by TEM. **G**, **H** Immunohistochemical analysis of Ki67 expression (**G**) and podoplanin-positive vessels (**H**) in tumors isolated from footpad (*n* = 5). **I** Relative levels of lncAKR1C2 in mouse serum exosomes (*n* = 5). **J** Relative levels of CPT1A mRNA in the lymph nodes (*n* = 5). **K** WB analysis of Flag-pep-AKR1C2 and CPT1A in lymph nodes (*n* = 5). ** indicates p < 0.01 and *** indicates p < 0.001.

To further examine the effect of exosomal lncAKR1C2 on LN metastasis, we established a popliteal lymphatic metastasis model as described previously [33, 36]. MKN45 cells treated as above were implanted in the footpads of nude mice, and the tumors and popliteal LNs were excised at the 40th day (Fig. 7A). As is shown by In Vivo Imaging System, overexpressed lncAKR1C2 markedly promoted lymphatic metastasis, while the knockdown of lncAKR1C2 dramatically suppressed LN metastasis and the growth of primary tumor (Fig. 7B, C). The highest volume and weight of LNs were also observed in the wild-type lncAKR1C2 group (Fig. 7D, E). Serum exosomes of mice were detected by TEM, and the image is shown in Fig. 7F. Consistent with the in vitro experiments, wild-type lncAKR1C2, but not the mutant, enhanced tumorigenesis and lymphangiogenesis of gastric cancer (Fig. 7G, H). In terms of mechanism, upregulation of exo-lncAKR1C2 promotes the expression of CPT1A in lymph nodes by encoding a microprotein (Fig. 7I–K). Therefore, these results provide in vivo evidence that the GC-exo-lncAKR1C2 encoded microprotein promotes lymphatic metastasis.

### In vivo role of lncAKR1C2 in tumorigenesis

**Fig. 8:**
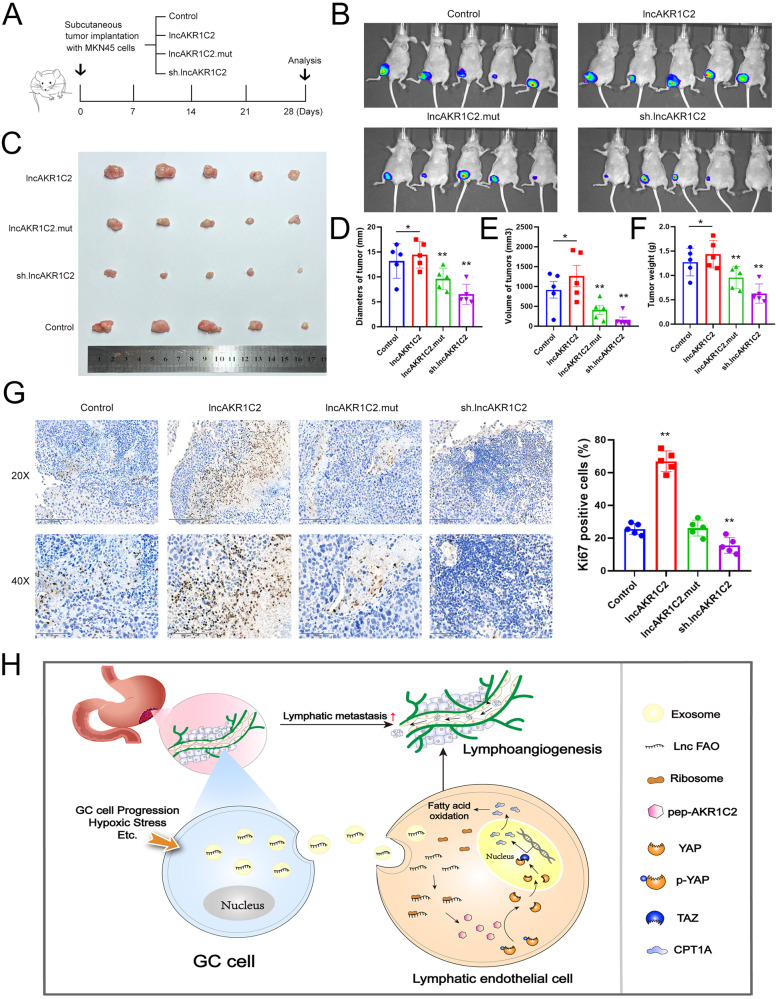
Lnc-AKR1C2 promotes tumor growth of gastric cancer in vivo. **A** Schematic description of the experimental design used to establish the animal model. (B, C) Histogram analysis (B) and images (C) of tumors in each group (*n* = 5). (D-F) Diameters (D), volumes (E) and weights (F) of tumors isolated from mice (*n* = 5). G. Immunohistochemical analysis of Ki67 in tumors described above (*n* = 5). H. A proposed model illustrating the role of the microprotein encoded by GC derived exo-lncAKR1C2 in regulating lipid metabolism of lymph endothelial cells. * indicates p < 0.05 and ** indicates p < 0.01.

Tumorigenesis and tumor burden are closely linked with lymphangiogenesis and lymphatic metastasis in various tumors, including gastric cancer [37, 38], and we investigated the in vivo role of lncAKR1C2 by using a subcutaneous xenograft model. MKN45 cells were infected with lentiviruses to overexpress wild-type or mutant lncAKR1C2, or knock down with lncAKR1C2, and these cells were then used for tumor implantation in mice (Fig. 8A). It was clearly shown that wild-type lncAKR1C2, but not the mutant, enhanced tumor growth, while downregulation of lncAKR1C2 suppressed tumorigenesis in a mouse model (Fig. 8B, C). Tumors in the lncAKR1C2 group also had larger diameters, volumes and weights (Fig. 8D–F). Higher levels of the proliferation marker Ki67 were also observed in the WT lncAKR1C2 group, while knockdown of lncAKR1C2 led to a sharp decrease in Ki67 levels, and there was no notable difference between the mutant and control groups (Fig. 8G). In summary, these results provide in vivo evidence for lncAKR1C2 mediated tumorigenicity and tumor growth.

Finally, we provide a schematic diagram to show the biological role of the microprotein encoded by GC secreted lncAKR1C2 in promoting lipid metabolism of lymph endothelial cells and lymphatic metastasis (Fig. 8H).

## Discussion

The relationship between tumor lymphangiogenesis and lymph node metastasis is well known, however, few studies have been focused on the metabolic characteristics of lymphatic endothelial cells. As a form of energy metabolism, the crucial role of β-fatty acid oxidation in tumor has been gradually recognized [39, 40], and a recent study suggests that lymphatic metastasis of melanoma requires that tumor cells undergo a metabolic shift towards FAO [14]. The current study initially showed the metabolic characteristics of LN metastases of gastric cancer by using comparative metabolomics analyses, and demonstrated that lipid metabolism plays a dominant role in the process of LNM of gastric cancer.

Exosome delivered lncRNAs and circRNAs are involved in tumorigenicity and tumor development in various cancers, but most studies on the mechanisms of exosomal lncRNAs or circRNAs focus on their role as ceRNAs or their effects on gene expression by regulating mRNA transcription [33, 41, 42]. The current study found that LNM-related exosomal lncAKR1C2 contains an sORF by using translation omics analysis, and provided in vitro and in vivo evidence that secreted lncAKR1C2 can directly encode a short a microprotein in lymphatic endothelial cells. Therefore, we found for the first time that exosome lncRNA can encode microproteins with biological functions in recipient cells.

Traditionally, the Hippo pathway contains the YAP/TAZ complex, and is subject to a phosphorylation cascade, in which MST1/2 phosphorylates the LATS1/2 kinase, while the phosphorylated LATS1/2 controls the phosphorylation of YAP/TAZ protein. The unphosphorylated YAP is translocated into the nucleus to enhance the expression of downstream genes [43, 44]. Our results demonstrated that the microprotein encoded by lncAKR1C2 interacts with YAP and decreases the levels of YAP phosphorylation, facilitating YAP translocation into the nucleus and CPT1A expression. Although YAP is required in LN metastasis of melanoma [14], the role of YAP in lymph endothelial cells has not been reported yet, and our study enriches the functional and molecular basis of the Hippo pathway.

Targeting proto-oncogenes in exosomes or inhibiting the secretion of these cancer-promoting ingredients are two common approaches to translational research. We have proven that suppression of lncAKR1C2 contributes to the prevention of lymphatic metastasis of gastric cancer by using a mouse model. Future studies focused on the biological process of exosome mediated lncAKR1C2 sorting and encapsulation should be conducted to develop techniques and methods to inhibit lncAKR1C2 secretion.

In summary, the microprotein encoded by GC derived exo-lncAKR1C2 promoted lymphangiogenesis and LN metastasis by regulating YAP phosphorylation and CPT1A expression, which depended on a metabolic shift towards fatty acid oxidation in lymph endothelial cells. Selective suppression of lymphatic metastasis may reduce the dissemination of cancer cells to distant organs at the initial development stage. Our findings provide insights for the study of exosomal delivered noncoding RNAs and highlight the diagnostic and therapeutic potential of targeting lncAKR1C2 in gastric cancer.

## Materials and methods

### Human samples

The tumor tissue samples and serum samples of GC patients were obtained from Tianjin Medical University Cancer Institute and Hospital. Sixty-one GC patients without LNM (LNM-) and ninety-four GC patients with LNM (LNM + ) were enrolled in the study. Written informed consent was provided by all patients, and the Ethics Committee of Tianjin Medical University Cancer Institute and Hospital approved all aspects of this study (Approval number: Ek2021002).

### Animals

Male nude mice (BALB/c-nu, 4–6 weeks, 18–20 g) were housed in a pathogen free animal facility with access to water and food, and allowed to eat and drink ad libitum. All of the experimental procedures were performed in accordance with protocols approved by the Institutional Animal Care and Research Advisory Committee of Tianjin Medical University Cancer Institute and Hospital (Approval number: NSFC-AE-2021002).

### Cell lines and cell culture

The human gastric cancer cell lines, HGC27 cells and MKN45 cells were purchased from Cell Bank of the Chinese Academy of Sciences (Shanghai, China), and were cultured in DMEM medium (Gibco, USA) supplemented with 10% fetal bovine serum. Human lymphatic endothelial cells (HLECS) were cultured in ECM (Endothelial Culture Medium) supplemented with 5% foetal bovine serum and 10% endothelial cell growth factor (ECGF). All cells were maintained at 37 °C in a 5% CO2 humidified atmosphere and tested for mycoplasma contamination before use.

### Isolation of exosomes

According to previous studies, exosomes in medium and in serum were isolated according to a previously described method [45, 46]. First, cells and other debris were removed by centrifugation at 300×g and 3000×g respectively, and then, the large-sized shedding vesicles were removed by centrifugation at 10,000×g for 30 min from the supernatant. Finally, exosomes were contained in the pellet by centrifugation at 110,000×g for 70 min from the supernatant and suspended in PBS. All steps were performed at 4 °C.

### Transmission electron microscopy (TEM) assay

Exosomes were fixed in 2.5% glutaraldehyde at pH 7.2 at 4 °C overnight. The samples were washed in PBS buffer 3 times (10 min each time) and then fixed in 1% osmium tetroxide at room temperature for 60 min. The samples were prepared as follows: the samples were embedded in 10% gelatine, fixed in glutaraldehyde at 4 °C and cut into several blocks (less than 1 mm). Then, dehydration of the samples was performed in increasing concentrations of alcohol (30%, 50%, 70%, 90%, 95%, and 100%×3, 10 min, each step). After that, infiltration of the samples was performed with increasing concentrations of Quetol-812 epoxy resin mixed with propylene oxide (25%, 50%, 75%, and 100%, 3 h, each step). Finally, the samples were embedded in pure, fresh Quetol-812 epoxy resin and polymerized at 35 °C for 12 h, 45 °C for 12 h, and 60 °C for 24 h. Ultrathin sections (100 nm) were cut using a Leica UC6 ultramicrotome and then stained with uranyl acetate for 10 min and lead citrate for 5 min at room temperature. An FEI Tecnai T20 transmission electron microscope was used to observe the samples.

### Nanoparticle tracking analysis (NTA)

The NanoSight NS 300 system (NanoSight Technology, Malvern, UK) was used to track the size and density of exosomes. The exosomes were resuspended in PBS at a concentration of 5 μg/mL and further diluted 100-500-fold to achieve 20–100 objects per frame. Samples were manually injected into the sample chamber at ambient temperature. Each sample was configured with a 488 nm laser and then measured in triplicate by a high-sensitivity sCMOS camera at camera setting 13 with an acquisition time of 30 s and a detection threshold setting of 7. At least 200 completed tracks were analysed per video, and then NTA analytical software (version 2.3) was used to analyse the data.

### PKH26 staining for exosomes

The PKH26 Red Fluorescent Cell Linker Kit (Sigma) was utilized for exosome staining. Fifty micrograms of exosomes (quantified by mass concentration with a NanoDrop 2000 Spectrophotometer, Thermo, Waltham, MA, USA) resuspended in 100 μL of diluent C was mixed with 100 μL of PKH26 dye solution (4 × 10^−6^M) and incubated for 1–5 min, which was stopped by adding 200 μL of serum. The labelled exosomes were then washed twice with PBS and coincubated with recipient cells in one well of a 6-well plate for 2–24 h before imaging was performed.

### Mass spectrum analysis

In the current study, LC-MS/MS analysis was performed on a Q Exactive mass spectrometer (Thermo Scientific) coupled to Easy nLC (Thermo Fisher Scientific). The mass spectrometer was used in positive ion mode, and the other mode settings were as follows: automatic gain control (AGC) target was set to 3e6, maximum injection time to 10 ms, and dynamic exclusion duration to 40.0 s. The resolution of survey scans was set to 70,000 at m/z 200, and the resolution of HCD spectra was set to 17,500 at m/z 200. The isolation width of the mass spectrometer was set to 2 m/z. The normalized collision energy was 30 eV, and the underfill ratio was specified as 0.1%. The instrument was run with peptide recognition mode enabled. Finally, the MS data were acquired by dynamically choosing the most abundant precursor ions dynamically from the survey scan (300–1800 m/z) for HCD fragmentation. The MS data were analysed by MaxQuant software version 1.5.3.17 (Max Planck Institute of Biochemistry, Martinsried, Germany).

### Fatty Acid (FA) quantification

Cell lines with different treatments were pretreated with oleic acid (OA, 200 μM) for 24 h and then cultured in low glucose media RPMI 1640 for an additional 48 h. Then, cells were stained with BODIPY-493/503 (dyes of fatty acids, 1 μg/mL) for 30 min and DAPI (1:1000 diluted by PBS) for 10 min. Finally, all groups were imaged using confocal microscopy (Zeiss, Jena, Germany).

### ATP quantification

Cells were seeded in 96-well plates, and control wells were also prepared to obtain background luminescence. A CellTiter-Glo Luminescent Cell Viability Assay (Promega, Madison, WI, USA) was used for ATP measurement. The plate and its contents were stored at RT for approximately 30 min. Then, 100 μL of CellTiter-Glo reagent was added to each well, followed by mixing for 2 min on a shaker to induce cell lysis and incubation at RT for 10 min to stabilize the luminescent signal. Finally, the luminescence was recorded via a microplate reader (BioTek Synergy H1, Winooski, VT, USA).

### Western blotting analysis

The protein levels of CPT1A, Flag-pep-AKR1C2, pep-AKR1C2, YAP, p127-YAP, VEGFC and HIF-1α were assessed by western blotting analysis and samples were normalized to GAPDH. For animals, mice were firstly sacrificed with carbon dioxide asphyxiation. Protein extraction was blocked with PBS-5% fat-free dried milk at room temperature for 1 h and incubated at 4 °C overnight with anti-CPT1A (1:1000; Abcam, Cambridge, UK; ab234111), anti-Flag (1:5,000; Abcam, Cambridge, UK; ab205606), anti-YAP (1:1000, Abcam; ab205270), anti p127-YAP (1:1000, Abcam; ab76252), anti-pep-AKR1C2 (1:1000, CST, 13035 S), anti-VEGFC (1:1000, Abcam; ab9546), anti-HIF-1α (Santa Cruz, sc-13515) anti-TSG101 (1:200; Santa Cruz, sc-7964), anti-Alix (1:1000, Abcam, ab275377), anti-CD9 (1:1000, Abcam, ab223052), anti-H3 (1:1000, Abcam, ab1791) and anti-GAPDH (1:3000, Santa Cruz, sc-365062) antibodies respectively.

### RNA isolation and quantitative RT-PCR

Total RNA was extracted from the cultured cells and tissues using TRIzol Reagent (Invitrogen) according to the manufacturer’s instructions. For animals, mice were firstly sacrificed with carbon dioxide asphyxiation. A total of 1000 ng of RNA from cultured cells or tissues was used for reverse transcription PCR (Eppendorf AG 22331 Hamburg, Germany). Then, 1 μL of cDNA was added to the real-time qPCR system. Relative levels of genes were calculated with equation the 2^-ΔCT^, in which ΔC_T_ = C_T gene_-C_T_ control.

### Ribosome footprint (RF) profiling (Ribo-seq) and RNA-Seq analyses

MKN45 cells were utilized for Ribo-seq and RNA-seq. Cells were pretreated with 2 μg/ml harringtonine and 100 μg/ml cycloheximide, washed with prechilled 1X PBS, and scraped for subsequent Ribo-seq. Read counts in ORFs were calculated, and the translation levels were normalized by the fragments per kilobase of transcript per million mapped reads (FPKM) method. Only ORFs with FPKM ≥ 1 in at least one sample group were retained for further analysis. Comparing HCT116/L-OHP and HCT116 cells, significantly differentially translated lncRNA ORFs were screened through fold change (FC) ≥ 2 and false discovery rate (FDR) < 0.05 using the edgeR package (http://www.r-project.org/). To evaluate the coding potential of the noncanonical ORFs, two methods, the ribosome release score (RRS) (Guttman et al., 2013) and ORF score (Bazzini et al., 2014), were applied. RF is the footprint left by the binding of ribosomal complexes to RNA. This binding may be a random combination without an effective translation process, or it may be a momentary footprint left by ribosomal complexes sliding over a translated RNA. Translated RF signals should conform to a three-base rhythm. In other words, during the translation process of RNA, ribosomes pause every codon of three bases, and the signal is concentrated in the main reading frame of an RNA ORF sequence. The ORF score is a method to determine whether the RF signal in an ORF conforms to the three-base rhythm. The 5th percentile of the ORF score from known protein-coding genes was chosen as the threshold to identify actively translated noncanonical ORFs. Furthermore, ribosomes are released from RNA upon encountering a stop codon, so the signal of RF at the 3’UTR after the stop codon is significantly lower than that at the CDS region. RRS is a method that is used to calculate the translation ratio between the CDS region and the 3’UTR. For noncanonical ORFs, the 3’UTR was defined as the region between the stop codon and the next possible start codon (in any frame). The calculation of RRS followed the formula:

RRS=log_2_((Ribo_FPKM(CDS)/Ribo_FPKM(30UTR))/(RNA_FPKM(CDS)/RNA_FPKM(30UTR))). All of the sequencing and bioinformatics analyses were performed by Gene Denovo Biotechnology Co., Ltd. (Guangzhou, China).

### Immunoprecipitation

Immunoprecipitation using anti-Flag antibody was performed at 48 h or 72 h after treatment. Cells were lysed in the lysis buffer containing 150 mM KCl, 25 mM Tris-HCl, pH 7.4, 5 mM EDTA, 0.5% Triton X-100, 5 mM dithiothreitol (DTT), PMSF and cocktail. The supernatant was mixed with anti-Flag antibody overnight at 4 °C, and then cocultured with beads (Santa Cruz) for 2–4 h at RT. The beads were washed five times in lysis buffer followed by western blotting (WB) analysis.

### Immunofluorescence

Cells were cultured on four-well chamber slides. At the time of harvest, cells were fixed with 4% paraformaldehyde and then permeabilized with 0.01% Triton X-100 for 10 min. Then cells were treated with anti-YAP (sc-101199) and anti-Flag (Abcam, ab205606) antibodies. In addition, all samples were treated with 40, 6-diamidino-2-phenylindole dye for nuclear staining (358 nm). For confocal microscopy, a Nikon C2 Plus confocal microscope was used.

### Immunohistochemistry (IHC)

The tumors were fixed in 4% paraformaldehyde, embedded in paraffin, sectioned and then stained with anti-VEGFC (Abcam, ab9546), anti-podoplanin (CST, 26981 and Abcam, ab256559) and anti-Ki67 (Abcam, ab15580) antibodies. For animals, mice were firstly sacrificed with carbon dioxide asphyxiation. Quantitative analysis was conducted by quantifying the fluorescence intensity from at least three or four sections.

### Establishment of the LNM model in nude mice

BALB/c nude mice (4–6 weeks old, 18–20 g) were used for the lymphatic metastasis model. Luciferase-labelled MKN45 cells (5 × 10^6^) were inoculated into the footpads or inguinal regions of the mice. The footpad tumors and popliteal LNs were excised when the tumors were 200 mm^3^ (LN volume (mm ^3^) = (length [mm] × width [mm]^2^) /2).

### Data analysis and statistical methods

All experiments were performed three times, and all quantitative data are presented as the mean value ± standard deviation (s.d.). Statistical tests were performed using Student’s t-test. No samples were excluded from the analysis. Values of p < 0.05 were considered significant. * indicates p < 0.05; ** indicates p < 0.01 and *** indicates p < 0.001.

### Supplementary information

Supplemental Material

## Data Availability

Data used to support the findings of this study are available from the corresponding author.

## References

[1] Sung H, Ferlay J, Siegel RL, Laversanne M, Soerjomataram I, Jemal A (2021). Global Cancer Statistics 2020: GLOBOCAN Estimates of Incidence and Mortality Worldwide for 36 Cancers in 185 Countries. CA Cancer J Clin.

[2] Kim DH, Choi MG, Noh JH, Sohn TS, Bae JM, Kim S (2015). Clinical significance of skip lymph node metastasis in gastric cancer patients. Eur J Surg Oncol.

[3] Deng JY, Liang H (2014). Clinical significance of lymph node metastasis in gastric cancer. World J Gastroenterol.

[4] Kosuga T, Konishi T, Kubota T, Shoda K, Konishi H, Shiozaki A (2019). Clinical significance of neutrophil-to-lymphocyte ratio as a predictor of lymph node metastasis in gastric cancer. BMC Cancer.

[5] Vos EL, Nakauchi M, Gonen M, Castellanos JA, Biondi A, Coit DG (2023). Risk of Lymph Node Metastasis in T1b Gastric Cancer: An International Comprehensive Analysis from the Global Gastric Group (G3) Alliance. Ann Surg.

[6] Pan S, An W, Tan Y, Chen Q, Liu P, Xu H (2021). Prediction model of lymph node metastasis risk in elderly patients with early gastric cancer before endoscopic resection: a retrospective analysis based on international multicenter data. J Cancer.

[7] Khalayleh H, Kim YW, Yoon HM, Ryu KW (2021). Assessment of lymph node metastasis in patients with gastric cancer to identify those suitable for middle segmental gastrectomy. JAMA Netw Open.

[8] Zhang C, Xie M, Zhang Y, Zhang X, Feng C, Wu Z (2022). Determination of survival of gastric cancer patients with distant lymph node metastasis using prealbumin level and prothrombin time: contour plots based on random survival forest algorithm on high-dimensionality clinical and laboratory datasets. J Gastric Cancer.

[9] Gershenwald JE, Ross MI (2007). Is sentinel-node biopsy superior to nodal observation in melanoma?. Nat Clin Pract Oncol.

[10] Morton DL, Thompson JF, Cochran AJ, Mozzillo N, Elashoff R, Essner R (2006). Sentinel-node biopsy or nodal observation in melanoma. N Engl J Med.

[11] Carracedo A, Cantley LC, Pandolfi PP (2013). Cancer metabolism: fatty acid oxidation in the limelight. Nat Rev Cancer.

[12] Cao YH (2019). Adipocyte and lipid metabolism in cancer drug resistance. J Clin Investig.

[13] Luo XJ, Cheng C, Tan ZQ, Li NM, Tang M, Yang LF (2017). Emerging roles of lipid metabolism in cancer metastasis. Mol Cancer.

[14] Lee CK, Jeong SH, Jang C, Bae H, Kim YH, Park I (2019). Tumor metastasis to lymph nodes requires YAP-dependent metabolic adaptation. Science.

[15] van Weverwijk A, Koundouros N, Iravani M, Ashenden M, Gao Q, Poulogiannis G (2019). Metabolic adaptability in metastatic breast cancer by AKR1B10-dependent balancing of glycolysis and fatty acid oxidation. Nat Commun.

[16] Wang LQ, Li CF, Song YM, Yan ZK (2020). Inhibition of carnitine palmitoyl transferase 1A-induced fatty acid oxidation suppresses cell progression in gastric cancer. Arch Biochem Biophys.

[17] Han S, Wei R, Zhang X, Jiang N, Fan M, Huang JH (2019). CPT1A/2-mediated FAO enhancement-a metabolic target in radioresistant breast cancer. Front Oncol.

[18] Wang YN, Zeng ZL, Lu J, Wang Y, Liu ZX, He MM (2018). CPT1A-mediated fatty acid oxidation promotes colorectal cancer cell metastasis by inhibiting anoikis. Oncogene.

[19] Xiong Y, Liu Z, Zhao X, Ruan S, Zhang X, Wang S (2018). CPT1A regulates breast cancer-associated lymphangiogenesis via VEGF signaling. Biomed Pharmacother.

[20] Oatman N, Dasgupta N, Arora P, Choi K, Gawali MV, Gupta N (2021). Mechanisms of stearoyl CoA desaturase inhibitor sensitivity and acquired resistance in cancer. Sci Adv.

[21] Sun L, Cai J, Gonzalez FJ (2021). The role of farnesoid X receptor in metabolic diseases, and gastrointestinal and liver cancer. Nat Rev Gastroenterol Hepatol.

[22] Crivelli SM, Giovagnoni C, Zhu ZH, Tripathi P, Elsherbini A, Quadri Z (2022). Function of ceramide transfer protein for biogenesis and sphingolipid composition of extracellular vesicles. J Extracell Vesicles.

[23] Thery C, Zitvogel L, Amigorena S (2002). Exosomes: composition, biogenesis and function. Nat Rev Immunol.

[24] Zhang H, Deng T, Liu R, Bai M, Zhou L, Wang X (2017). Exosome-delivered EGFR regulates liver microenvironment to promote gastric cancer liver metastasis. Nat Commun.

[25] Zhang H, Deng T, Liu R, Ning T, Yang H, Liu D (2020). CAF secreted miR-522 suppresses ferroptosis and promotes acquired chemo-resistance in gastric cancer. Mol Cancer.

[26] Zhang HY, Deng T, Ge SH, Liu Y, Bai M, Zhu KG (2019). Exosome circRNA secreted from adipocytes promotes the growth of hepatocellular carcinoma by targeting deubiquitination-related USP7. Oncogene.

[27] Zhang M, Zhao K, Xu X, Yang Y, Yan S, Wei P (2018). A peptide encoded by circular form of LINC-PINT suppresses oncogenic transcriptional elongation in glioblastoma. Nat Commun.

[28] Wang Y, Wu S, Zhu X, Zhang L, Deng J, Li F (2020). LncRNA-encoded polypeptide ASRPS inhibits triple-negative breast cancer angiogenesis. J Exp Med.

[29] Khyzha N, Khor M, DiStefano PV, Wang L, Matic L, Hedin U (2019). Regulation of CCL2 expression in human vascular endothelial cells by a neighboring divergently transcribed long noncoding RNA. Proc Natl Acad Sci USA.

[30] Pirlog R, Calin GA (2022). KRAS mutations as essential promoters of lymphangiogenesis via extracellular vesicles in pancreatic cancer. J Clin Invest.

[31] Wang Y, Zhang W, Liu W, Huang L, Li D, Wang G (2021). Long Noncoding RNA VESTAR regulates lymphangiogenesis and lymph node metastasis of esophageal squamous cell carcinoma by enhancing VEGFC mRNA stability. Cancer Res.

[32] Kong Y, Li Y, Luo Y, Zhu J, Zheng H, Gao B (2020). circNFIB1 inhibits lymphangiogenesis and lymphatic metastasis via the miR-486-5p/PIK3R1/VEGF-C axis in pancreatic cancer. Mol Cancer.

[33] Chen C, Luo Y, He W, Zhao Y, Kong Y, Liu H (2020). Exosomal long noncoding RNA LNMAT2 promotes lymphatic metastasis in bladder cancer. J Clin Invest.

[34] Wang M, Yu W, Cao X, Gu H, Huang J, Wu C (2022). Exosomal CD44 Transmits Lymph Node Metastatic Capacity Between Gastric Cancer Cells via YAP-CPT1A-Mediated FAO Reprogramming. Front Oncol.

[35] Wang M, Zhao X, Qiu R, Gong Z, Huang F, Yu W (2021). Lymph node metastasis-derived gastric cancer cells educate bone marrow-derived mesenchymal stem cells via YAP signaling activation by exosomal Wnt5a. Oncogene.

[36] Chen C, He W, Huang J, Wang B, Li H, Cai Q (2018). LNMAT1 promotes lymphatic metastasis of bladder cancer via CCL2 dependent macrophage recruitment. Nat Commun.

[37] Karaman S, Detmar M (2014). Mechanisms of lymphatic metastasis. J Clin Invest.

[38] Rault-Petit B, Do Cao C, Guyetant S, Guimbaud R, Rohmer V, Julie C (2019). Current management and predictive factors of lymph node metastasis of appendix neuroendocrine tumors: a national study from the french group of endocrine tumors (GTE). Ann Surg.

[39] Wang X, Song H, Liang J, Jia Y, Zhang Y (2022). Abnormal expression of HADH, an enzyme of fatty acid oxidation, affects tumor development and prognosis (Review). Mol Med Rep.

[40] Choi KM, Kim JJ, Yoo J, Kim KS, Gu Y, Eom J (2022). The interferon-inducible protein viperin controls cancer metabolic reprogramming to enhance cancer progression. J Clin Invest.

[41] Liu TY, Han CC, Fang PQ, Ma ZF, Wang XX, Chen H (2022). Cancer-associated fibroblast-specific lncRNA LINC01614 enhances glutamine uptake in lung adenocarcinoma. J Hematol Oncol.

[42] Gao J, Ao YQ, Zhang LX, Deng J, Wang S, Wang HK (2022). Exosomal circZNF451 restrains anti-PD1 treatment in lung adenocarcinoma via polarizing macrophages by complexing with TRIM56 and FXR1. J Exp Clin Cancer Res.

[43] Nishina H (2022). Physiological and pathological roles of the Hippo-YAP/TAZ signaling pathway in liver formation, homeostasis, and tumorigenesis. Cancer Sci.

[44] Xie JH, Wang YX, Ai D, Yao L, Jiang HF (2022). The role of the Hippo pathway in heart disease. Febs J.

[45] Quah BJ, O’Neill HC (2005). The immunogenicity of dendritic cell-derived exosomes. Blood Cells Mol Dis.

[46] Valadi H, Ekstrom K, Bossios A, Sjostrand M, Lee JJ, Lotvall JO (2007). Exosome-mediated transfer of mRNAs and microRNAs is a novel mechanism of genetic exchange between cells. Nat Cell Biol.

