# Supplementary material for: The microprotein encoded by exosomal lncAKR1C2 promotes gastric cancer lymph node metastasis by regulating fatty acid metabolism
Source: Cell Death Dis. 2023 Oct 30;14(10):708. doi: 10.1038/s41419-023-06220-1 (PMC10616111; doi:10.1038/s41419-023-06220-1)
Supplement: Supplementary file 1 — Supplemental Material [file 41419_2023_6220_MOESM1_ESM.pdf]

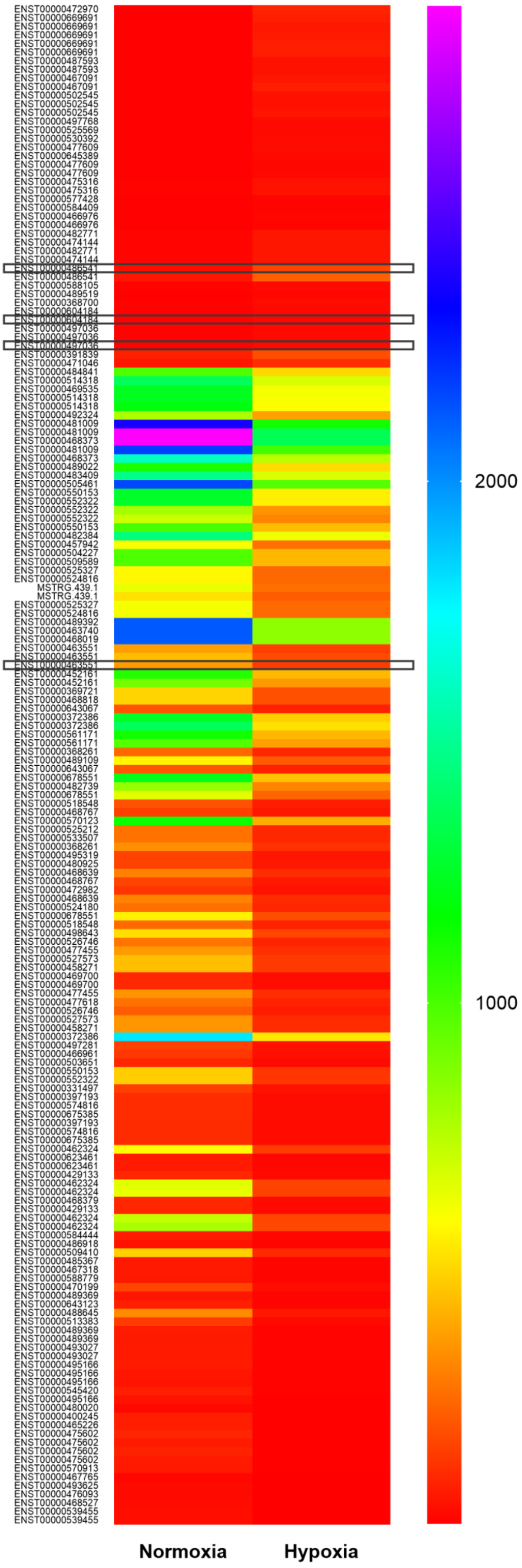
ENST00000472970
ENST00000669691
ENST00000669691
ENST00000669691
ENST00000669691
ENST00000669691
ENST00000487593
ENST00000487593
ENST00000467091
ENST00000467091
ENST00000502545
ENST00000502545
ENST00000502545
ENST00000497768
ENST00000525569
ENST00000530392
ENST00000477609
ENST00000645389
ENST00000477609
ENST00000477609
ENST00000475316
ENST00000475316
ENST00000577428
ENST00000584409
ENST00000466976
ENST00000466976
ENST00000482771
ENST00000474144
ENST00000482771
ENST00000474144
ENST00000486541
ENST00000486541
ENST00000588105
ENST00000489519
ENST00000368700
ENST00000604184
ENST00000604184
ENST00000497036
ENST00000497036
ENST00000497036
ENST00000391839
ENST00000471046
ENST00000484841
ENST00000514318
ENST00000469535
ENST00000514318
ENST00000514318
ENST00000492324
ENST00000481009
ENST00000481009
ENST00000468373
ENST00000481009
ENST00000468373
ENST00000489022
ENST00000483409
ENST00000505461
ENST00000550153
ENST00000552322
ENST00000552322
ENST00000552322
ENST00000550153
ENST00000482384
ENST00000457942
ENST00000504227
ENST00000509589
ENST00000525327
ENST00000524816
MSTRG.439.1
MSTRG.439.1
ENST00000525327
ENST00000524816
ENST00000489392
ENST00000463740
ENST00000468019
ENST00000463551
ENST00000463551
ENST00000463551
ENST00000452161
ENST00000452161
ENST00000369721
ENST00000468818
ENST00000643067
ENST00000372386
ENST00000372386
ENST00000561171
ENST00000561171
ENST00000368261
ENST00000489109
ENST00000643067
ENST00000678551
ENST00000482739
ENST00000678551
ENST00000518548
ENST00000468767
ENST00000570123
ENST00000525212
ENST00000533507
ENST00000368261
ENST00000495319
ENST00000480925
ENST00000468639
ENST00000468767
ENST00000472982
ENST00000468639
ENST00000524180
ENST00000678551
ENST00000518548
ENST00000498643
ENST00000526746
ENST00000477455
ENST00000527573
ENST00000458271
ENST00000469700
ENST00000469700
ENST00000477455
ENST00000477618
ENST00000526746
ENST00000527573
ENST00000458271
ENST00000372386
ENST00000497281
ENST00000466961
ENST00000503651
ENST00000550153
ENST00000552322
ENST00000331497
ENST00000397193
ENST00000574816
ENST00000675385
ENST00000397193
ENST00000574816
ENST00000675385
ENST00000462324
ENST00000623461
ENST00000623461
ENST00000429133
ENST00000462324
ENST00000462324
ENST00000468379
ENST00000429133
ENST00000462324
ENST00000462324
ENST00000584444
ENST00000486918
ENST00000509410
ENST00000485367
ENST00000467318
ENST00000588779
ENST00000470199
ENST00000489369
ENST00000643123
ENST00000488645
ENST00000513383
ENST00000489369
ENST00000489369
ENST00000493027
ENST00000493027
ENST00000495166
ENST00000495166
ENST00000495166
ENST00000545420
ENST00000495166
ENST00000480020
ENST00000400245
ENST00000465226
ENST00000475602
ENST00000475602
ENST00000475602
ENST00000475602
ENST00000570913
ENST00000467765
ENST00000493625
ENST00000476093
ENST00000468527
ENST00000539455
ENST00000539455
2000
1000
Normoxia
Hypoxia

Figure S1. The full heatmap of 176 lncORFs screened out by synthetically using multiple scoring systems.

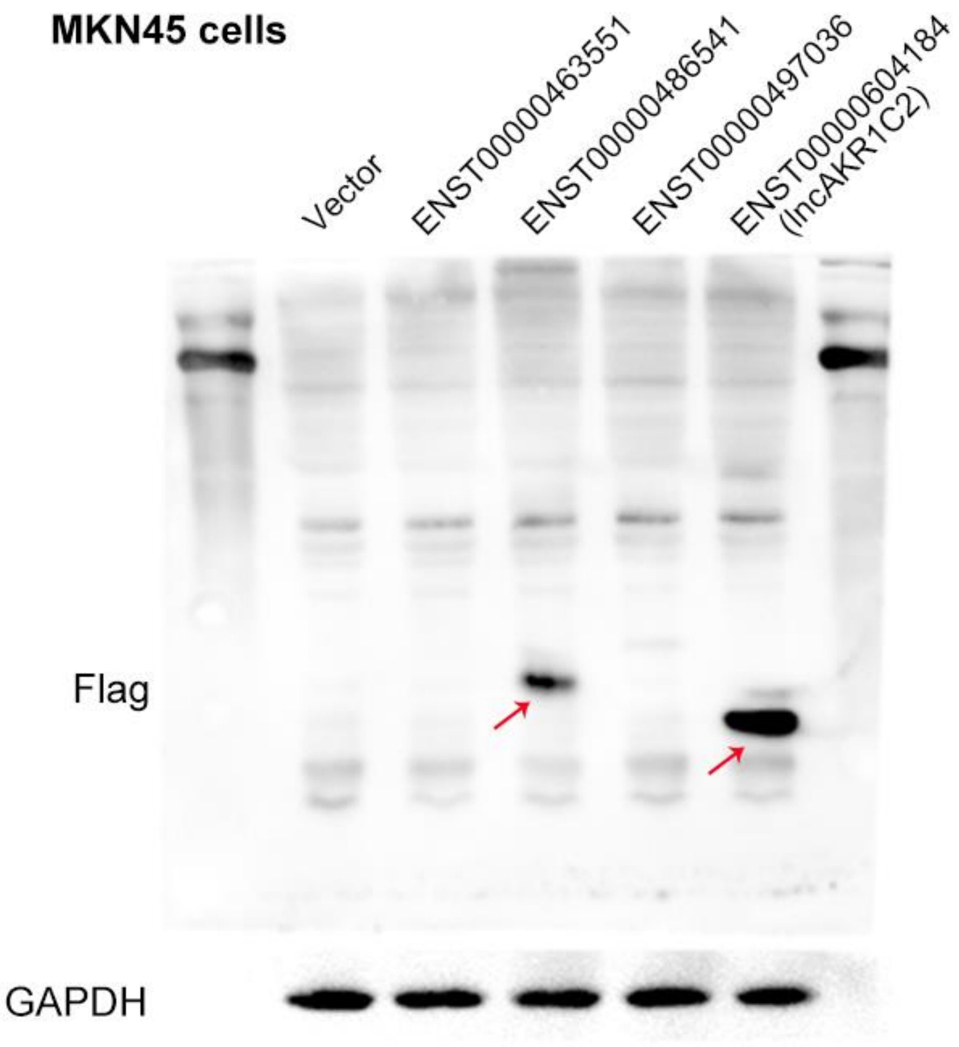

Figure S2. WB analysis of the Flag fusion peptides encoded by the four selected lncORFs. Four lncRNAs, ENST00000463551, ENST00000486541, ENST00000497036 and ENST00000604184, were selected and the plasmids containing the full length of the lncRNAs and the Flag-tags were constructed. The ability of these lncRNAs to encode short peptides was tested by WB analysis using anti-Flag antibody.

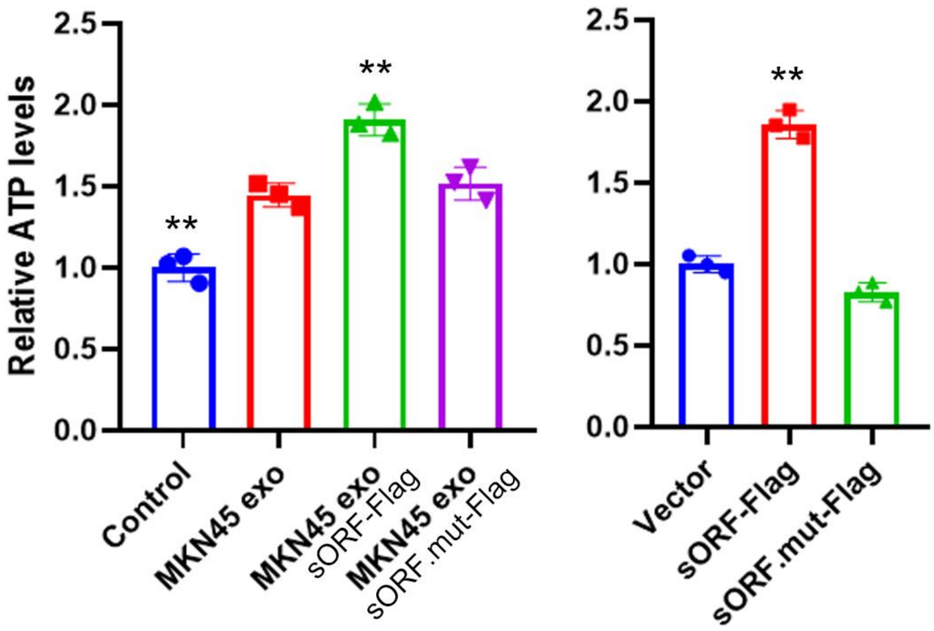

Figure S3. Relative ATP levels of HLECS cells. HLECS cells were treated with saline, MKN45 exos, MKN45 exos sORF-Flag and MKN45 exos sORF.mut-Flag, or transfected with sORF-Flag, sORF.mut-Flag and control vector, and ATP levels were detected in each group (n=3). ** indicates p<0.01.

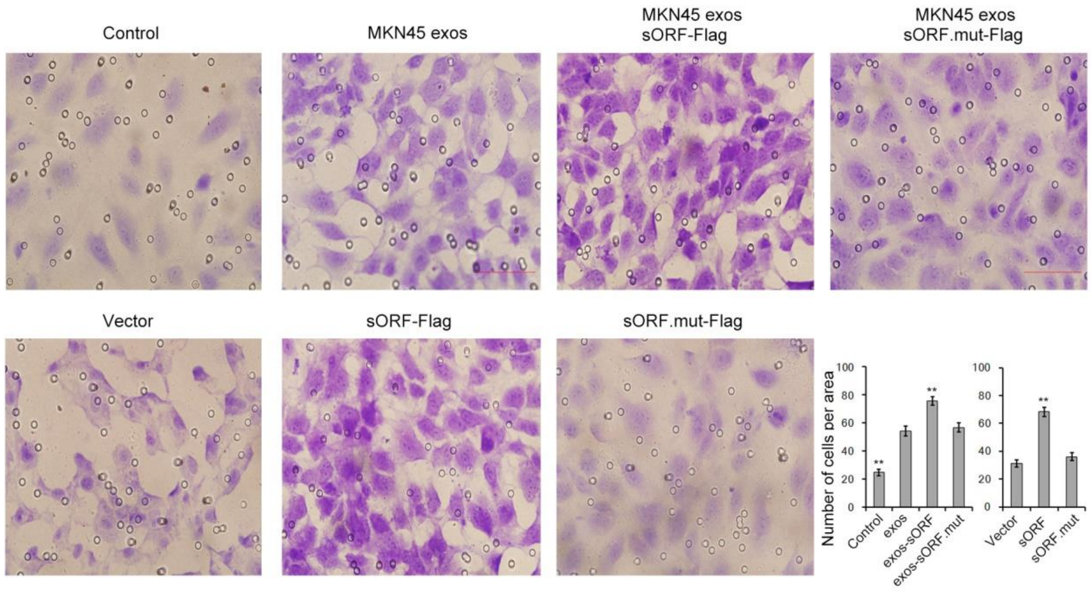

Figure S4. Transwell analysis of HLECS cells in each group (n=3). ** indicates p<0.01.

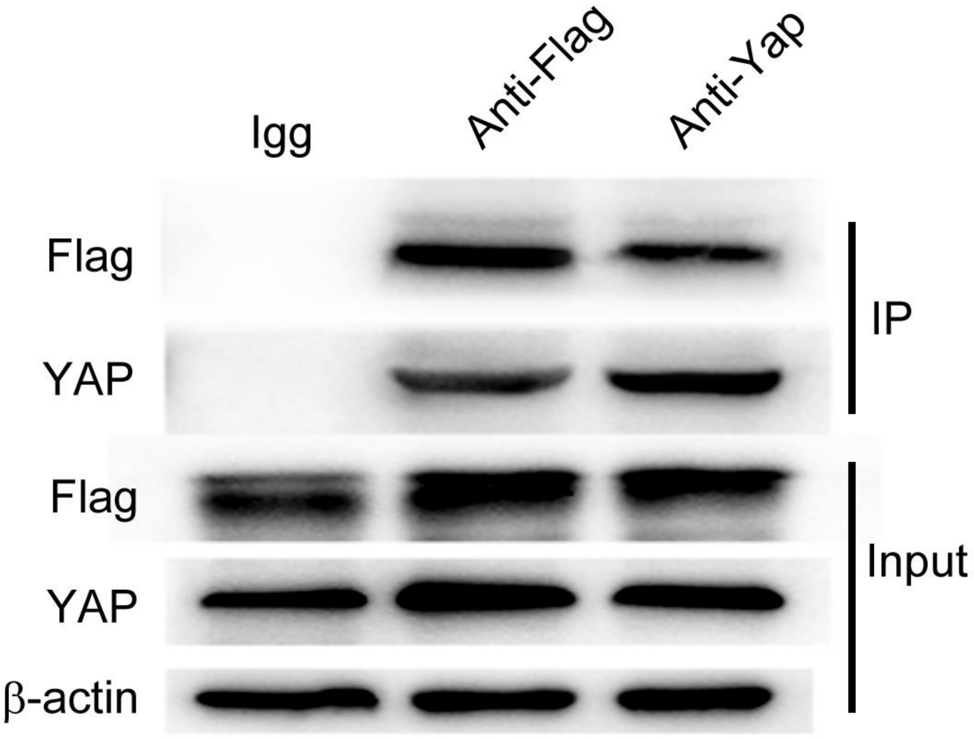

Figure S5. Verification of the direct interaction of YAP and pep-AKR1C2. Flag-labeled pep-AKR1C2 was overexpressed in HLECS cells, and the combination of co-IP and WB analysis was performed by using anti-Flag and anti-YAP antibodies respectively.

Extended Table 1. Mass spectrum analysis of the proteins interacted with Flag-labeled pep-AKR1C2 in HLECS cells.

**Protein names**	**Flag**	**IgG**
Elongation factor 1-delta	6.129430216	-6.12943022
cDNA FLJ55805	6.018258535	-6.01825854
Cytoskeleton-associated protein 4	6.000880014	-6.00088001
Elongation factor 1-beta	6.000880014	-6.00088001
F-actin-capping protein subunit alpha-1	5.946081655	-5.94608165
Yes-associated protein 1	5.799258905	-5.79925891
Serpin B4	5.725863134	-5.72586313
Protein ZNRD2	5.725863134	-5.72586313
F-actin-capping protein subunit alpha-2	5.699638962	-5.69963896
Heterogeneous nuclear ribonucleoproteins A2/B1	5.699638962	-5.69963896
Elongation factor 1-gamma	5.672425342	-5.67242534
Keratin, type I cuticular Ha1	5.644144671	-5.64414467
Tropomyosin alpha-4 chain	5.644144671	-5.64414467
40S ribosomal protein S16	5.614709844	-5.61470984
40S ribosomal protein S3	5.614709844	-5.61470984
Uncharacterized protein	5.584022634	-5.58402263
Keratin, type I cuticular Ha3-II	5.518430223	-5.51843022
Centrosomal protein 43	5.518430223	-5.51843022
Signal sequence receptor subunit delta	5.483252726	-5.48325273
Endoplasmic reticulum chaperone BiP	5.446271408	-5.44627141
Microsomal glutathione S-transferase 1	5.446271408	-5.44627141
60S ribosomal protein L23a	5.446271408	-5.44627141
Peptidase A1 domain-containing protein	5.366083713	-5.36608371
Erlin-2	5.322378796	-5.3223788
Tubulin alpha chain	5.322378796	-5.3223788
Bleomycin hydrolase	5.275854131	-5.27585413

Large tumor suppressor 1	5.22612062	-5.22612062
Q9Y5K6	5.172702623	-5.17270262
P62820	5.172702623	-5.17270262
Lamina-associated polypeptide 2	5.172702623	-5.17270262
Q9Y2W1	5.115010218	-5.11501022
Very-long-chain (3R)-3-hydroxyacyl-CoA dehydratase 3	5.115010218	-5.11501022
Cofilin	5.115010218	-5.11501022
Heterogeneous nuclear ribonucleoprotein M	5.115010218	-5.11501022
RNA-binding motif protein	5.052299377	-5.05229938
2-phospho-D-glycerate hydro-lyase	5.052299377	-5.05229938
Malate dehydrogenase	4.983613129	-4.98361313
Serine/arginine-rich splicing factor 7	4.983613129	-4.98361313
Polyadenylate-binding protein 1	4.983613129	-4.98361313
60S acidic ribosomal protein P2	2.360498567	-2.36049857
Protein S100-A14	2.31503893	-2.31503893
FLJ00144 protein	2.31503893	-2.31503893
60S ribosomal protein L30	2.31503893	-2.31503893
Thioredoxin	2.189324482	-2.18932448
Peptidyl-prolyl cis-trans isomerase	2.078185492	-2.07818549
Protein S100-A9	1.951205086	-1.95120509
60S ribosomal protein L22	1.874113117	-1.87411312
Cystatin-A	1.859901045	-1.85990104
Zymogen granule protein 16 homolog B	1.846899496	-1.8468995
60S ribosomal protein L24	1.843596882	-1.84359688
Erlin-1	1.843596882	-1.84359688
L-lactate dehydrogenase	1.843596882	-1.84359688
Protein S100-A8	1.818618826	-1.81861883
Ubiquitin-40S ribosomal protein S27a	1.790281104	-1.7902811

60S ribosomal protein L7	1.785904476	-1.78590448
Protein S100-A16	1.726445869	-1.72644587
Dermcidin	1.711498995	-1.71149899
Lysozyme C	1.711498995	-1.71149899
RNA transcription, translation and transport factor protein	1.654507388	-1.65450739
Proteasome subunit beta type-1	1.654507388	-1.65450739
P05121	1.654507388	-1.65450739
Keratin, type I cytoskeletal 19	-5.322378796	5.3223788
cDNA FLJ58539	-5.322378796	5.3223788
